# Molecular Mechanism of *Puerarin* Against Diabetes and its Complications

**DOI:** 10.3389/fphar.2021.780419

**Published:** 2022-01-04

**Authors:** Yi-ling Bai, Ling-ling Han, Jun-hui Qian, Hao-zhong Wang

**Affiliations:** ^1^ College of Basic Medicine, Chengdu University of Traditional Chinese Medicine, Chengdu, China; ^2^ Affiliated Hospital of Chengdu University of Traditional Chinese Medicine, Chengdu, China

**Keywords:** diabetes, diabetes complications, puerarin, hypoglycemic mechanism, protection mechanisms

## Abstract

*Puerarin* is a predominant component of *Radix Puerarin*. Despite its anti-tumor and anti-virus effects and efficacy in improving cardiovascular or cerebrovascular diseases and preventing osteoporosis, it has been shown to protect against diabetes and its complications. This review summarizes the current knowledge on *Puerarin* in diabetes and related complications, aiming to provide an overview of antidiabetic mechanisms of *Puerarin* and new targets for treatment.

## Introduction

Diabetes is a non-communicable metabolic disease characterized by chronic hyperglycemia. It has become the third epidemic following cardiovascular diseases and tumors (Meng et al., 2017; Niu et al., 2017). The International Diabetes Federation (IDF) reported 463 million diabetic adults worldwide, and this figure is projected to reach 700 million by 2045 (Nanditha et al., 2016; Yan et al., 2018). Significant and persistent hyperglycemia can lead to dysfunction in various cell types (Wang C et al., 2020), efficiently inducing complications such as nephropathy, retinopathy, angiocardiopathy, cerebrovascular diseases, and neuropathy (Fletcher et al., 2007; Cheng et al., 2015; Srivali et al., 2015; Cusi et al., 2017; Azmi et al., 2019). Diabetes treatment alongside a strict diet and exercise control exerts a pronounced effect on blood glucose control. However, undesirable side effects such as hypoglycemia, gastrointestinal reactions, liver damage, and lactic acidosis brought by antidiabetic agents are particularly concerned (Srivali et al., 2015), which are the crux of implementing patient compliance and result in unsatisfactory management of complications.

As the demand for and use of traditional Chinese medicine (TCM) continues to rise globally, remarkable antidiabetic effects and safety of TCM products have been reported. Among them, *ge-gen* in Chinese or *Radix Puerariae* (RP) is a powerful healing herb (sweet and cool) widely used in ancient China (Prasain et al., 2003; Zhou et al., 2014). According to *Sheng Nong’s Herbal Classic* during the Han Dynasty, it has the effects of relieving restless thirst, vomiting, and stiffness, and pain in joints and clearing internal heat by enriching *yin*. Thus, various toxicity is relieved (Huang et al., 2018; Wei et al., 2020). It also has the action of reducing fever, producing fluid, and relieving diarrhea due to spleen deficiency (Zhi et al., 2018). RP has been reported for diabetes treatment for two thousand years, as evidenced by ancient records regarding TCMs (e.g., *Yu Quan* [jade-spring] pill, *Xiao Ke* [relieving-thirst] pill, *Qiwei Baizhu* [seven-ingredient] powder) for restless thirst. Overall, RP-related TCMs for hyperglycemia frequently used in ancient China showed a good effect on diabetes and its complications (Cao et al., 2006; Zan, 2010; Cai et al., 2014; Liu J et al., 2014; Wong et al., 2015; Zhao et al., 2015). The isoflavone *Puerarin* is the affective component of RP (Cao et al., 2006), which has been shown to protect against various pathophysiological processes, including angiocardiopathy, osteoporosis, inflammation, liver damage, cancers (Jiang, 2004), and diabetes (Wong et al., 2011). However, RP in diabetes treatment only received scant attention from the rest of the world. We aimed to provide an overview of the roles of *Puerarin* in protection against diabetes and related complications for better knowledge of RP in diabetes treatment.

Six electronic databases, including China National Knowledge Infrastructure (CNKI), Wanfang database, Chinese Scientific Journals Database (VIP), PubMed, EMBASE, and Cochrane Library, were searched from June 2001 to June 2021 for identifying eligible studies. No restriction on language or publication status was imposed. The following terms were used in a combination for the electronic search: *Kudzu root*, *Kudzu*, *Pueraria Mirifica*, *Pueraria lobata*, *Puerarin*, diabetes, diabetes complications, complications of diabetes, diabetic nephropathy, diabetic cardiomyopathy, diabetic retinopathy, diabetic macroangiopathy, diabetic peripheral neuropathy, DN, NC, DR, DM, DPN, randomized control, randomization, randomized clinical trials, RCT, and trails. Inclusion criteria were animal studies and possible signaling pathways, including protective effects against diabetic complications. A third reviewer solved any inconsistency. Manual searches were performed to identify relevant studies in the reference lists of the included studies.

## Hypoglycemic Mechanism of *Puerarin*

It is generally accepted that insulin resistance (IR) and defective β-cell secretions are the main links in the pathogenesis of diabetes and the mechanisms involved in regulating blood glucose through four organs: pancreas, liver, skeletal muscle, and adipose tissue. We attempted to explore the molecular mechanisms of glucose-lowering by *Puerarin* acting on the above four target organs.

### Pancreas

The endocrine function of the pancreas is performed by the islets, which are the center of controlling the dynamic balance of blood glucose in the body and are an essential endocrine organ in the pancreatic tissue. Among them, β-cell is the primary cell of the islets and mainly secrete insulin. If insulin production is insufficient, or if IR occurs, it may lead to elevated blood glucose. *Puerarin* acts on the following molecular mechanisms of the pancreas to lower blood glucose.

#### Enhance GLP-1R Signaling Pathway

A recent report showed that chronic hyperglycemia could lead to the loss of the glucagon-like peptide-1 receptor (GLP-1R) from the cell surface and impairment of GLP-1R signaling (Yang et al., 2016a). Therefore, recovery of the GLP-1R expression itself and the GLP-1R signaling transduction might be a strategy for diabetic treatment (Tomita, 2016).

**FIGURE 1:**
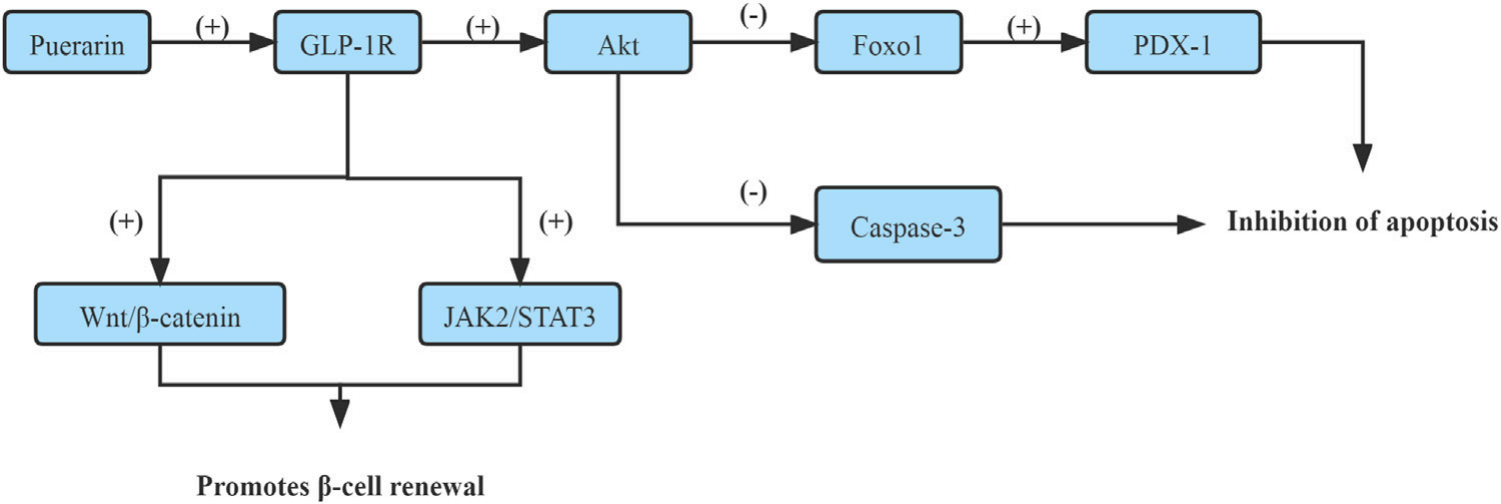
Enhance GLP-1R signaling pathway.

On one side, it has been shown that *Puerarin* rescued the β-cell failure and promoted β-cell proliferation through up-regulating GLP-1R expression, which enhanced GLP-1R signaling and activated its downstream target protein kinase B (Akt), which led to the inactivation of forkhead box transcription factor O1 (Foxo1) and Caspase-3 subsequently. Foxo1 acts as a transcription factor to inhibit pancreatic duodenum homeobox-1 (PDX-1) activity and mediate-cell dysfunction and apoptosis (Yang et al., 2016a). The caspase family of proteins is involved in inducing apoptosis (Liang et al., 2019). (Figure 1)

On the other hand, *Puerarin* induced β-cell replication and neogenesis in pancreatic ductal cells of HFD mice depended on GLP-1R expression in ductal cells together with activating β-catenin and STAT3, subsequently activated Wnt/β-catenin and JAK2/STAT3, up-regulation of PDX-1 and Ngn3 expression, which up-regulation of TCFTL2 expression, that might be downstream effectors of the GLP-1R signaling cascade. *Puerarin* triggers the pancreatic ductal epithelial cell to β-cell conversion through activating GLP-1R/Wnt/STAT3 signaling cascade (Wang T et al., 2020). (Figure 1)

#### Reduce the Generation of ROS

As is known, mitochondrial oxidative stress is a crucial factor contributing to IR and β-cell dysfunction. Excess reactive oxygen species (ROS) could activate downstream apoptotic factors, including cytochrome C (Cyt-C) and AIF, and induce β-cell apoptosis (Liang et al., 2019).

As revealed by some study, *Puerarin* significantly decreased ROS generation, which might be mediated via increasing gene expression of ROS scavengers-MnSod and Gpx-1 (Li et al., 2014), subsequently up-regulated the anti-oxidant superoxide dismutase 2 (SOD2) and Gpx-1, and the anti-apoptotic β-cell lymphoma-2 (Bcl-2), and decreased the pro-apoptotic Bcl-2-associated X (Bax), thus, reducted of oxidative stress to protects telomere length in pancreatic β-cell of diabetic rats, inhibited pancreatic β-cell apoptosis as evidenced (Li et al., 2014; Chen et al., 2017). Another mechanism is to decrease mitochondrial malondialdehyde (MDA) levels and increase superoxide dismutase (SOD) levels in the pancreas by reducing ROS production, thus restoring the Na, K- or Ca^2+^-ATPase activity to protect the pancreas (Sun et al., 2011).

#### Improve Caspase/AIF/Apoptosis Pathway

The caspase family proteins increase the mitochondrial permeability, which then triggers Cyt-C release from the mitochondria. AIF is also a crucial factor responsible for mitochondrial apoptosis. Therefore, the Caspase/AIF/apoptosis pathway may be a new target of *Puerarin* for diabetes mellitus therapy (Liang et al., 2019).

**FIGURE 2:**
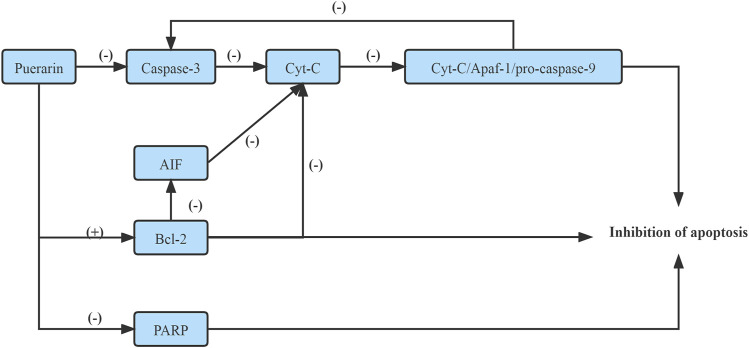
Improve Caspase/AIF/apoptosis pathway.

It has been shown that *Puerarin* inhibited the mitochondrial permeability by down-regulating the expression of the caspase family proteins, which inhibited Cyt-C release from the mitochondria and inhibited the formation of apoptotic bodies, namely, Cyt-C/Apaf-1/pro-caspase-9 complex, which inhibited the expression of Caspase-3, realized the purpose of preventing apoptosis of pancreatic cell apoptosis (Liang et al., 2019). On the other hand, *Puerarin* prevented pancreatic cell apoptosis by inducing the activation of Bcl-2, a regulatory factor of AIF(Liang et al., 2019). Moreover, *Puerarin* suppressed the activation of apoptosis-related proteins, including poly ADP-ribose polymerase (PARP) and Caspase-3, subsequently inhibiting β-cell apoptosis (Li et al., 2014). (Figure 2)

#### Improve PI3K/Akt Signaling Pathway

**FIGURE 3:**
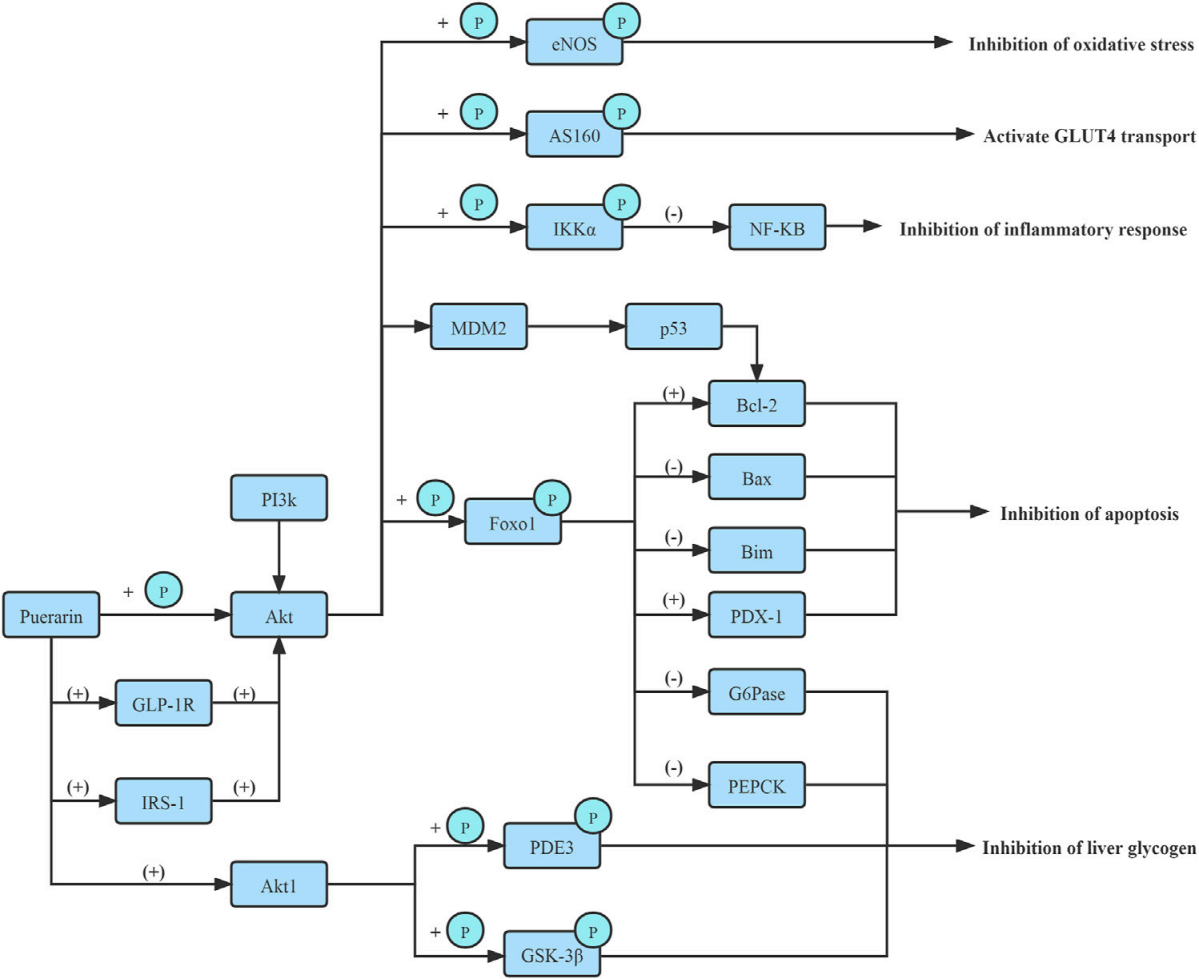
Improve PI3K/Akt signaling pathway.

The phosphoinositide 3-kinase (PI3K)/Akt signaling pathway regulates β-cell function and survival. *Puerarin* rapidly activated AKT phosphorylation and protected pancreatic β-cell survival by the PI3K/Akt signaling pathway, increasing pancreatic β-cell mass via β-cell apoptosis inhibition in diabetic mice (Li et al., 2014). (Figure 3)

Furthermore, *Puerarin* promoted β-EP synthesis in pancreatic tissue and activated pancreatic β-cell opioid receptors, which promoted insulin secretion (Chen et al., 2010a). Another mechanism is to inhibit the UCP2 gene expression via up-regulating sirtuins1 (SIRT1) and AMP-Activated Protein Kinase (AMPK) protein expressions to protect pancreatic β-cell (Xiong et al., 2006; Chen et al., 2017).

Taken together, *Puerarin* increased β-cell mass and promoted β-cell survival through up-regulating GLP-1R expression, inhibiting ROS or Caspase/AIF/apoptosis pathway, increasing PI3K/Akt signaling pathway, which enhanced insulin receptor signaling and inhibited oxidative stress and β-cell apoptosis in the pancreas, subsequently, elevated serum insulin and improved IR, thus, lowered fasting blood glucose (FBG) and glycated hemoglobin (HbA1c) levels. The hypoglycemic mechanism of *Pueraria* acting on the pancreas is shown in Table 1.

**TABLE 1:** The hypoglycemic mechanism of *Pueraria* acting on the pancreas.

Model	Assay	Species	Dose	Effect	Pathways (Target cells)	Target organs	References
High-fat diet (HFD)	*In vitro*	Male C57BL/6 mice (4 weeks)	Puerarin 150 mg/kg for 35 d	Puerarin promotes pancreatic β-cell survival	GLP-1R signaling pathway (GLP-1R)	Pancreas	Yang et al. (2016a)
High-fat diet (HFD)	*In vitro*	Mice db/db (male, 4 weeks)	Puerarin 150 mg/kg/d for 55 d	Puerarin promotes pancreatic β-cell survival	GLP-1R signaling pathway (GLP-1R)	Pancreas	Yang et al. (2016a)
Diabetes induced by STZ + High-fat diet (HFD)	*In vivo*	Male Kunming mice (18–22 g)	Puerarin 80 mg/kg for 15 days (i.g.)	Puerarin prevents apoptosis of pancreatic cell apoptosis	Caspase/AIF/apoptosis signaling pathway (Bcl-2 and the caspase family proteins)	Pancreas	Liang et al. (2019)
High-fat diet (HFD)	*In vivo*	Male C57BL/6 mice (5 weeks)	Puerarin 150, 300 mg/kg/d for 20 d	Puerarin induced β-cell replication and neogenesis in pancreatic ductal cells of HFD.	GLP-1R/Wnt/STAT3 signaling pathway (GLP-1R)	Pancreas	Wang T et al. (2020)
Diabetes induced by STZ	*In vivo*	Male C57BL/6 mice (8 weeks, 20–22 g)	Puerarin 100 mg/kg for 3 days before STZ (i.p.)	Puerarin acts directly on pancreatic β-celll-protecting function and survival and protects pancreatic islet survival by preventing β-cell apoptosis	Reduce the generation of ROS	Pancreas	Li et al. (2014)
					Caspase/AIF/apoptosis signaling pathway (PARP and Caspase-3); PI3K/Akt signaling pathway (Akt)		
CoCl2	*In vitro*	Mouse insulinoma MIN6 cells (passage 22–30)	Puerarin 0.1, 1, 10 mM for 8 h	Puerarin acts directly on pancreatic β-celll-protecting function and survival and protects pancreatic islet survival by preventing β-cell apoptosis	Reduce the generation of ROS	Pancreas	Li et al. (2014)
					Caspase/AIF/apoptosis signaling pathway (PARP and Caspase-3); PI3K/Akt signaling pathway (Akt)		
Diabetes induced by STZ + High-fat diet (HFD)	*In vivo*	Male SD rats (160–180 g)	Puerarin 80 mg/kg for 4 weeks (i.p.)	Puerarin exerts preventive and remedial effects on the diabetic pancreatic β-cell, which is probably due to protecting telomere length and inhibiting β-cell apoptosis via alleviating oxidative damage	Reduce the generation of ROS; SIRT1/AMPK signaling pathway	Pancreas	Chen et al. (2017)
Proteoxypyrimidine solution	*In vivo*	Wistar rats (8 weeks, 180–240 g)	Puerarin 80 mg/kg/d (i.p., n = 10)	Puerarin protects the pancreas	Reduce the generation of ROS	Pancreas	Sun et al. (2011)
Diabetes induced by STZ	*In vivo*	Male SD rats (6 weeks, 180–220 g)	Puerarin 80, 120, 160 mg/kg for 12 weeks	Puerarin promoted β-EP synthesis in pancreatic tissue and activated pancreatic β-cell opioid receptors, which promoted insulin secretion	opioid receptors signaling pathway (opioid receptors)	Pancreas	Chen et al. (2010a)
500 mM H2O2	*In vitro*	Male Wistar rats pancreatic islets	Puerarin 100 mM for 48 h	Puerarin protects the pancreas	SIRT1/AMPK signaling pathway	Pancreas	Xiong et al. (2006)

### Skeletal Muscle and Adipose Tissue

The skeletal muscle is the significant tissue of glucose metabolism, accounting for nearly 75% of the whole-body insulin-stimulated glucose uptake. IR in skeletal muscle is a critical component of the etiology of diabetes (Chen et al., 2018a). As the largest endocrine organ in the body, adipose tissue secretes various protein substances that regulate blood glucose metabolism and are essential regulators of human glucose homeostasis. *Puerarin* regulates blood glucose metabolism in skeletal muscle and adipose tissue through the following molecular mechanisms.

#### Activate GLUT4 Transmission

Glucose transport, which depends on insulin-stimulated translocation of glucose carriers to the cell membrane, is the rate-limiting step in carbohydrate metabolism of skeletal muscle and adipose tissue. The subtype 4 form is predominant in skeletal muscle and adipose tissue. It is possible that *Puerarin* can enhance glucose uptake and improve IR via increasing glucose transporter 4 (GLUT4) mRNA and protein expressions (Hsu et al., 2003).

Numerous studies have reported that *Puerarin* increased GLUT4 mRNA and protein expressions on the plasma membrane in skeletal muscle and adipocyte membrane via improved insulin signaling protein, namely, protein kinase B (PKB/Akt), pronouncedly reduced IR and enhanced glucose absorption from blood circulation and lower blood sugar levels (Hsu et al., 2003; Song and Bi, 2004; Xu et al., 2005).

Moreover, *Puerarin* increased serum β-EP content via activating opioid μ-receptor in adipocytes and skeletal muscle cell membrane, subsequently activating the phospholipase C-protein kinase C (PLC-PKC) pathway up-regulated the GLUT4 mRNA expression, thus promoting glucose uptake in adipose tissue and skeletal muscle tissue (Chen et al., 2010a).

In the skeletal muscle, insulin receptor signaling is achieved through insulin receptor substrate-1 (IRS-1), which coordinates PI3K-dependent activation of Akt. *In vitro* and *in vivo* experiments confirmed that *Puerarin* improved insulin signaling, namely, IRS-1, through activating μ-opioid receptor, subsequently activated Akt, and phosphorylated its substrate AS160, and promoted GLUT4 translocation and glucose uptake (Chen et al., 2018a).

In addition, *Puerarin* could markedly improve the insulin resistance of 3T3-L1 lipocyte, which is realized possibly by way of activating for GLUT4 exocytosis via Cb1 signaling, promoting GLUT4 transposition to the cell membrane to increase the transportation of glucose, and improving IR, thus increasing insulin sensitivity and lowering blood glucose (Zhao and Zhou, 2012). Therefore, *Puerarin* improved the GLUT4 content of adipocytes in the IR state and promoted the translocation of intimal GLUT4 to the outer membrane, thereby increasing the transport and utilization of adipocytes to glucose (Li and Bi, 2004).

**FIGURE 4:**
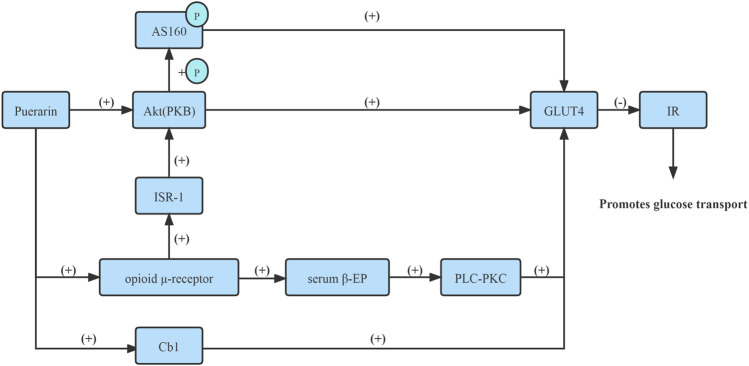
Activate GLUT4 transmission.

Together, *Puerarin* activated GLUT4 transmission, thus improving IR, thereby increasing the transport and utilization of skeletal muscle cells and adipocytes to glucose (Figure 4).

#### Activate PPAR Receptor Expressions

On one side, intramuscular peroxisome proliferator-activated receptors α (PPARα) activation promotes ingestion, utilization, and catabolism of fatty acids through activation of downstream genes (Wu et al., 2013). One of the main bio-functions of insulin receptors (InsR) promotes intracellular glucose uptake within target tissues and exerts a crucial physiological effect modulating glucose homeostasis. Therefore, *Puerarin* has been shown to up-regulate InsR and PPARα mRNA expressions in the gastrocnemius via stimulating phosphodiesterase 3 (PDE3) and insulin-like growth factor-1 (IGF-1) to enhance insulin signaling and receptor sensitivity (Wu et al., 2013), promoted glucose uptake.

On the other hand, peroxisome proliferators-activated receptors γ (PPARγ) is a ligand-activated nuclear transcription factor that is distributed in tissues with active energy metabolism, such as adipose and skeletal muscle and is mainly involved in the regulation of insulin sensitivity, adipocyte differentiation, and the expression of many genes related to glucolipid metabolism. The mechanism of *Puerarin* could activate PPARγ through stimulating PPARγ mRNA expression, and then increased the heterodimer of PPARγ and retinoic acid receptor (RXR), which can bind to response elements present in target genes activated by these transcription factors, subsequently potentiated preadipocyte differentiation, and improved IR and insulin sensitivity, which potentiated the glucose-uptake of adipocytes, *in vivo* and *in vitro* assays. Further investigations are needed to confirm this suggestion (Xu et al., 2005).

#### Promote Fatty Acid Oxidation

In addition, growing evidence suggested that mitochondrial dysfunction and the associated impairment of the oxidative capacity of skeletal muscle contribute to the development of insulin resistance. Thus, the improvement of mitochondrial function and fatty acid oxidation in muscle is regarded as a potential therapeutic approach for the treatment of diabetes (Chen et al., 2018b).

*In vivo* and *in vitro* assays, *Puerarin* protected mitochondria of skeletal muscle against oxidative damage via increasing sirtuins 3(SIRT3) and SOD2 expressions and suppressing p66Shc phosphorylation, which led to promoted the oxidation of fatty acids, which thus prevented the accumulation of intramyocellular lipids (IMCLs) in diabetic rats (Chen et al., 2018b). As for puerarin-mediated adipocyte IR inhibition, the administration of *Puerarin* significantly decreased membrane levels of fatty acid translocase (CD36) and increased the phosphorylation of AMPK and acetyl carboxylase (ACC) to enhance the activity of carnitine palmitoyltransferase-1b (CPT-1b), which thus reduced the uptake of fatty acids, promoted the transport of fatty acids into mitochondria for oxidation, and prevented the accumulation of IMCLs (Chen et al., 2018b). *In-vitro* experiments show that *Puerarin* fueled fatty acid oxidation in myotubes against lipid accumulation, which suppressed membrane CD36 levels and reduced IR (Chen et al., 2018b).

In conclusion, *Puerarin* could improve IR and enhance glucose uptake by increasing GLUT4 transport, activating PPAR receptor expressions, and promoting fatty acid oxidation in skeletal muscle cells and adipocytes, thus lowering blood sugar. The hypoglycemic mechanism of *Puerarin* acting on the skeletal muscle and adipose tissue is shown in Table 2.

**TABLE 2:** The hypoglycemic mechanism of *Puerarin* acting on the skeletal muscle and adipose tissue.

Model	Assay	Species	Dose	Effect	Pathways (Target cells)	Target organ	References
Diabetes induced by STZ	*In vivo*	Male SD rats (6 weeks, 180–220 g)	Puerarin 80, 120, 160 mg/kg for 12 weeks	Puerarin increases serum β-EP content and promotes the uptake of glucose by fat and skeletal muscle tissues	Activate GLUT4 transport (opioid μ-receptor)	Skeletal muscle and adipose tissue	Chen et al. (2010a)
High-fat diet (HFD)	*In vivo*	Male SD rats (160–180 g)	Puerarin 100 mg/kg for 4 weeks (i.p.)	Puerarin enhances μ-opioid receptor expression and phosphorylation and increases insulin-stimulated GUT4 translocation to the plasma membrane in the skeletal muscle of diabetic rats	Activate GLUT4 transport (opioid μ-receptor)	Skeletal muscle	Chen et al. (2018a)
0.75 mM of palmitate	*In vitro*	Rat L6 skeletal muscle cells	0.3 mM puerarin for 24 h	Puerarin affects insulin sensitivity in the muscle in a μ-opioid receptor-dependent manner *in vitro*	Activate GLUT4 transport (opioid μ-receptor)	Skeletal muscle	Chen et al. (2018a)
Diabetes induced by STZ	*In vitro*	Isolated soleus muscle of STZ-diabetic male Wistar rats (200–250 g)	Puerarin 0.01–100 mol/L	Puerarin enhances glucose uptake	Activate GLUT4 transport (PKB/Akt)	Skeletal muscle	Hsu et al. (2003)
Diabetes induced by STZ	*In vivo*	Male Wistar rats (200–250 g)	Puerarin 5–20 mg/kg/d (i.v., n = 8); 15 mg/kg (i.v., n = 8) three times daily for 3 days	Puerarin up-regulates GLUT-4 mRNA and protein expression in soleus muscle and enhances glucose uptake	Activate GLUT4 transport (PKB/Akt)	Skeletal muscle	Hsu et al. (2003)
High glucose	*In vitro*	Preadipocytes of male SD rats (100–150 g)	Puerarin 3, 10, 30 mol/L for 48 h	Puerarin can potentiate glucose uptake of insulin resistance adipocytes induced by high glucose treatment in a dose-dependent manner	Activate GLUT4 transport (PKB/Akt)	Adipose tissue	Xu et al. (2005)
DMEM	*In vitro*	HUVECs of male SD rats (100–150 g)	Puerarin 3, 10, 30 mol/L for 3 days	Puerarin potentiates insulin-induced preadipocyte differentiation, promotes glucose uptake of adipocytes that have been induced insulin resistance by high glucose	Activate GLUT4 transport (PKB/Akt); Activate PPAR receptor expressions (PPARγ)	Adipose tissue	Xu et al. (2005)
Hypercholesterolemic diet	*In vivo*	Male SD rats (150–200 g)	Puerarin 100 mg/kg/d for 4 weeks (i.p., n = 8)	Puerarin reduces blood glucose and insulin levels and up-regulates the protein expression of GLUT-4 in skeletal muscle	Activate GLUT4 transport (PKB/Akt)	Skeletal muscle	Song and Bi, (2004)
FFA	*In vitro*	Preadipocyte line 3T3-L1	Puerarin 1.5, 0.75 mg/ml for 48 h	Puerarin improves insulin resistance and increases the transportation of glucose of 3T3-L1 lipocytes	Activate GLUT4 transport (Cb1)	Adipose tissue	Zhao and Zhou, (2012)
High glucose + High-fat diet (HFD)	*In vivo*	Male Wistar rats (6 weeks, 165–180 g)	Puerarin 100 mg/kg for 6 weeks (i.p., n = 10)	Puerarin increases the transport and utilization of adipocytes to glucose	Activate GLUT4 transport	Adipose tissue	Li and Bi, (2004)
Diabetes induced by STZ	*In vivo*	Male BALB/c mice (180–200 g)	Puerarin 20, 40, 80 mg/kg for 14 h	Puerarin up-regulates the InsR, PPARa mRNA expressions of gastrocnemius in diabetic mice	Activate PPAR receptor expressions (PDE3 and IGF-1)	Skeletal muscle	Wu et al. (2013)
Diabetes induced by STZ + High-fat diet (HFD)	*In vivo*	Male SD rats (6 weeks)	Puerarin 100 mg/kg for 4 weeks (i.p.)	Puerarin effectively alleviates dyslipidemia and decreases the accumulation of intramyocellular lipids	Promote fatty acid oxidation (SIRT3 and SOD2)	Skeletal muscle	Chen et al. (2018b)
DMEM+ 0.75 mM palmitate	*In vitro*	Rat L6 skeletal muscle cells	Puerarin (0.3 mM) for 24 h	Puerarin effectively alleviates dyslipidemia and decreases the accumulation of intramyocellular lipids	Promote fatty acid oxidation (CD36)	Skeletal muscle	Chen et al. (2018b)

### Liver

The pathophysiological mechanism of elevated blood glucose involves a variety of tissues and cells, of which the liver has the most closed relationship with type 2 diabetes. The liver plays a crucial role in glucose metabolism. The regulation of glucose production and storage by the liver is crucial for gluconeogenesis and glycogen synthesis. The most important source of endogenous glucose production in hepatic gluconeogenesis, a hallmark in type 2 diabetes patients (Liu et al., 2021).

The PI3K/Akt signaling pathway is considered the foremost signal transduction pathway and plays a significant regulatory role in gluconeogenesis. Glucose-6-phosphatase (G6pase) and phosphoenolpyruvate carboxykinase (PEPCK) are the pivotal rate-limiting enzymes in gluconeogenesis, and Foxo1 modulates insulin sensitivity. Under the condition of insulin resistance, PI3K/Akt activity is decreased, and hepatic gluconeogenesis is promoted due to increased PEPCK and G6pase expression driven by Foxo1 (Liu et al., 2021).

*In vitro* and *in vivo* experiments confirm, *Puerarin* could enhance the phosphorylation of Foxo1 by activating PI3K/Akt signaling pathway in liver tissues of type 2 diabetes rates and elevating the pFoxo1/Foxo1 protein and mRNA expressions, and further inhibiting the expression of G6pase and PEPCK. Thus hepatic gluconeogenesis and endogenous glucose production in the liver are suppressed. Furthermore, *Puerarin* improved IR (Shen et al., 2019; Liu et al., 2021). (Figure 3)

Moreover, *in vitro*, the protein kinase Bα2 (Akt1) is predicted to be a target protein of *Puerarin*. *Puerarin* targeted the PH domain of Akt1, inhibited Akt1’s transmembrane effect, and activated Akt1 to improve the phosphorylation or activity of downstream proteins, such as glycogen synthase kinase-3β (GSK-3β) and PDE3, and ultimately regulated glucose and lipid metabolism (Shen et al., 2019). (Figure 3)

Consequently, *Puerarin* inhibits hepatic gluconeogenesis by activating PI3K/Akt signaling pathway. The hypoglycemic mechanism of *Pueraria* acting on the liver is shown in Table 3.

**TABLE 3:** The hypoglycemic mechanism of *Pueraria* acting on the liver.

Model	Assay	Species	Dose	Effect	Pathways (Target cells)	Target organ	References
Diabetes induced by STZ + High-fat diet (HFD)	*In vivo*	Male Wistar rats (160–200 g)	Puerarin 300 mg/kg/d for 4 weeks (p.o.)	Puerarin administration improves glucose tolerance and inhibits hepatic gluconeogenesis in T2DM rats	PI3K/Akt signaling pathway (Akt)	Liver	Liu et al. (2021)
PA	*In vitro*	HepG2	Puerarin 10, 100, 1,000 μmol/L	Puerarin administration improves glucose tolerance and inhibits hepatic gluconeogenesis in T2DM rats	PI3K/Akt signaling pathway (Akt)	Liver	Liu et al. (2021)
DMSO	*In vitro*	HepG2	Puerarin 10–5 mol/L for 24 h	Puerarin regulates glucose and lipid metabolism	PI3K/Akt signaling pathway (Akt1)	Liver	Shen et al. (2019)

Finally, *Puerarin* regulates systemic functions to lower blood sugar. *In vitro* and *in vivo* studies prove that the antidiabetic effects of *Puerarin* might be related to the inhibition of protein tyrosine phosphatase-1B.

(PTP1B), therefore, better the insulin signaling pathway and increased insulin receptor’s, achieving the purpose of moderating blood glucose levels by markedly boosting glucose uptake and escalating glucose tolerance (Sun et al., 2019). Moreover, *Puerarin* significantly increased plasma β-endorphin (β-EP) concentrations and reduced blood glucose levels in diabetic rats via within α1-adrenergic receptors (α1-AR) and adrenal medulla 1-adrenaline (1-A) activation (Chen et al., 2004). In addition, *Puerarin* decreased the level of blood glucose and aldose reductase activity in red blood cells, accordingly inhibiting the formation of glycation products and expression of AGE mRNA (Kim et al., 2006; Zhang et al., 2006; Zhang et al., 2009; Liu et al., 2018). See Table 4 for details.

**TABLE 4:** Hypoglycemic mechanism of *Pueraria* acting on systemic functions.

Model	Assay	Species	Dose	Effect	Pathways (Target cells)	Target organ	References
Insulin-resistant HepG2 cells	*In vitro*	Insulin-resistant HepG2 cells	Puerarin (0.0115, 0.0058, 0.0029, 0.0014 mg/ml)	Puerarin increases the insulin sensitivity in HepG2 cells and enhances the glucose uptake, and betters the insulin signaling pathway	Inhibition PTP1B (PTP1B)	Systemic function	Sun et al. (2019)
Diabetes induced by STZ	*In vivo*	Male C57BL/6 mice (4 weeks, 18–22 g)	Puerarin (0.25, 0.5, 1, 2 g/kg)	Puerarin escalates glucose tolerance	Inhibition PTP1B (PTP1B)	Systemic function	Sun et al. (2019)
Diabetes induced by STZ	*In vitro*	Male Wistar rats (200–250 g)	Puerarin 15 mg/kg (i.v., n = 8)	Puerarin reduces blood glucose level and enhanced plasma-endorphin level in the absence of insulin stimulation	Activate the 1-A or α1-AR-mediated signalings (1-A or α1-AR)	Systemic function	Chen et al. (2004)
d-galactose	*In vivo*	SD rats (6 weeks)	Puerarin 75, 150, 300 mg/kg for 6 weeks (i.g.)	Puerarin decreases the level of blood glucose and the activity of aldose reductase in red blood cells, inhibiting the formation of glycation products	Inhibition of AGEs formation	Systemic function	Zhang et al. (2006)

## Protective Mechanism of *Pueraria* Against Diabetic Complications

Diabetic complications are based on long-term chronic hyperglycemia, which damages small, medium, large, and microvessels and causes organ lesions. Chronic hyperglycemia, as a specific characterization of diabetes, has adverse effects on various organs such as the kidney, eyes, heart, and especially nervous, easily caused by diabetic nephropathy (DN), diabetic macroangiopathy (DM), diabetic retinopathy (DR), diabetic cardiomyopathy (DC), diabetic peripheral neuropathy (DPN) and so on. *Puerarin* has a protective effect against the above diabetic complications. The protective mechanism of *Puerarin* against diabetic complications is shown in Table 5.

**TABLE 5:** The protective mechanism of *Puerarin* against diabetic complications.

Model	Assay	Species	Dose	Protective mechanism	Pathways (Target cells)	Diabetes complications	References
Diabetes induced by STZ	*In vivo*	Male C57BL/6 mice (18–22 g)	Puerarin 20, 40, 80mg/kg/d for 8 weeks (i.g., n = 10)	Anti-oxidative stress; Anti-inflammatory	SIRT1/Foxo1 pathway (SIRT1); SIRT1-NF-κB pathway (SIRT1)	Diabetic Nephropathy	Xu et al. (2016)
Diabetes induced by STZ	*In vivo*	eNOS-null male mice on a C57BL/6 (8 weeks)	Puerarin 20 mg/kg/d for 8 weeks	Anti-oxidative stress; Anti-inflammatory	SIRT1-NF-κB pathway (SIRT1)	Diabetic Nephropathy	Li et al. (2017)
Normal glucose	*In vitro*	Murine podocytes	Puerarin for 24 h	Anti-oxidative stress; Anti-inflammatory	SIRT1-NF-κB pathway (SIRT1)	Diabetic Nephropathy	Li et al. (2017)
Diabetes induced by STZ	*In vivo*	SD rats (7–8 weeks, 180–220 g)	Puerarin 40, 80, 160 mg/kg/d for 8 weeks (i.p., n = 10)	Anti-inflammatory	NF-κB pathway (NF-kBp65)	Diabetic Nephropathy	Cui and Wang, (2010)
Diabetes induced by STZ	*In vivo*	Male SD rats (7 weeks, 200–250 g)	Puerarin 0.25, 0.5, 1mg/kg/d for 8 weeks (i.g., n = 10)	Anti-inflammatory	Inhibition ICAM-1 and TNF-a expressions	Diabetic Nephropathy	Pan et al. (2015)
Diabetes induced by STZ	*In vivo*	Male Wistar rats (210–230 g)	Puerarin 140, 200 mg/kg/d for 30 days (p.o., n = 10)	Anti-inflammatory	TGF-β1/Smad2 signal pathway (TGF-β1)	Diabetic Nephropathy	She et al. (2014)
High-fat diet (HFD)	*In vivo*	KKAy male mice (9–11 weeks, 25–28 g)	Puerarin 1.3 mg/kg/d for 24 weeks (n = 8)	Anti-inflammatory	Inhibit TGF-β1 and TGF-βRI expressions (α-SMA)	Diabetic Nephropathy	Yi et al. (2013)
Diabetes induced by STZ	*In vivo*	Male SD rats (8 weeks, 180–220 g)	Puerarin 20, 40, 80 mg/kg for 8 weeks (i.g., n = 9)	Anti-inflammatory	TLR4/MyD88/NF-kBp65 pathway (miRNA-140-5P)	Diabetic Nephropathy	Xu X et al. (2020)
DMEM-F12	*In vitro*	HK-2	Puerarin 80 mg/LSP for 48 h	Anti-inflammatory	TLR4/MyD88/NF-kBp65 pathway (miRNA-140-5P)	Diabetic Nephropathy	Xu X et al. (2020)
Diabetes induced by STZ	*In vivo*	C57BL/6 mouse (8 weeks, 18–22 g)	Puerarin 40, 80 mg/kg/d for 8 weeks (p.o., n = 10)	Promoted autophagy	PERK/eIF2α/ATF4 signaling pathway (PERP)	Diabetic Nephropathy	Xu Y et al. (2020)
Diabetes induced by STZ	*In vivo*	Male C57BL/6 mice (8 weeks)	Puerarin 5, 10, 20, 40 mg/kg for 12 weeks (n = 8)	Promoted autophagy	AMPK/SIRT1 pathway (SIRT1)	Diabetic Nephropathy	Li et al. (2020)
HG; DMEM	*In vitro*	Immortalized mouse podocytes	Puerarin 15 min	Promoted autophagy	AMPK/SIRT1 pathway (SIRT1)	Diabetic Nephropathy	Li et al. (2020)
Diabetes induced by STZ	*In vivo*	Male SD rats (200–250 g)	Puerarin 100 mg/kg/d for 8 weeks (i.p., n = 11)	Inhibit AGEs formation	Inhibit AGEs formation	Diabetic Nephropathy	Shen et al. (2009)
Diabetes induced by STZ	*In vivo*	Male SD rats (180–200 g)	Puerarin 100 mg/kg/d for 4 weeks (i.p., n = 10)	Anti-oxidative stress	Inhibit eNOS expressions	Diabetic Nephropathy	Zhang et al. (2015)
HG	*In vitro*	mMVEC	Puerarin 5, 10, 20 μM + HG for 24 h	Anti-inflammatory	NF-κB pathway (NF-kB)	Diabetic Macroangiopathy	Lian et al. (2019)
Diabetes induced by STZ	*In vivo*	Male SD rats (250–280 g)	Puerarin 15, 45 mg/kg/d for 3 weeks (i.p., n = 8)	Anti-inflammatory	NF-κB pathway (NF-kBp65)	Diabetic Macroangiopathy	Li et al. (2016)
L-DMEM	*In vitro*	HUVECs	Puerarin 1, 10, 50 μM for 8 h	Anti-inflammatory	IKKb/NF-kB pathway (NF-kBp65); IKKb/IRS-1 pathway	Diabetic Macroangiopathy	Huang et al. (2012)
DMSO	*In vitro*	EA.hy926 cells	Puerarin 100 μM for 1 h	Anti-oxidative stress	PI3K/Akt and CaMKII/AMPK pathway (IRS-1)	Diabetic Macroangiopathy	Hwang et al. (2011)
FBS	*In vitro*	Rat VSMCs	Puerarin 10–100 μM for 1 h	Anti-oxidative stress	PKCβ2/Rac1 pathway (Rac1)	Diabetic Macroangiopathy	Zhu et al. (2010)
Diabetes induced by STZ	*In vivo*	Male SD rats (8–10 weeks, 180–220 g)	Puerarin 80 mg/kg/d for 12 weeks (i.g., n = 25)	Inhibit AGEs formation	Inhibit AGEs formation	Diabetic Retinopathy	Liu et al. (2018)
Diabetes induced by STZ	*In vivo*	SD rats (8–10 weeks, 200–220 g)	Puerarin 250, 500 mg/kg (i.v., n = 10)	Anti-inflammatory	JAK2/STAT3 pathway	Diabetic Retinopathy	Cai et al. (2017)
Diabetes induced by STZ	*In vivo*	Male Wistar rats (6–8 weeks, 180–220 g)	Puerarin 25, 50, 100 mg/kg for 12 weeks (i.p., n = 18)	Anti-inflammatory	Nrf2/HO-1 pathway	Diabetic Retinopathy	Zhang and Li, (2019)
Diabetes induced by STZ	*In vivo*	Wistar rats (280–320 g)	Puerarin 100 mg/kg for 6 weeks (i.p., n = 20)	Anti-inflammatory	Nrf2/ERK pathway	Diabetic Retinopathy	Zhang and Wang, (2019)
Diabetes induced by STZ	*In vivo*	Male SD mice (5–6 weeks, 200–220 g)	Puerarin 140 mg/kg for 56 days (i.p., n = 36)	Anti-apoptosis	Fas/FasL pathway (ONOO-)	Diabetic Retinopathy	Hao et al. (2012)
Diabetes induced by STZ	*In vivo*	Male SD rats (250 g)	Puerarin 140 mg/kg for 56 days (i.p., n = 12)	Anti-apoptosis	Inhibit ONOO- expression	Diabetic Retinopathy	Hao et al. (2010)
ONOO-	*In vitro*	C57BL/6 mice RPE (Passage 2–3)	Puerarin for 24 h	Anti-apoptosis	Inhibit ONOO- expression	Diabetic Retinopathy	Hao et al. (2010)
Diabetes induced by STZ + HG + High-fat diet (HFD)	*In vivo*	Male SD rats (120–160 g)	Puerarin 0.56, 2.81 g/kg for 4 weeks (i.g.)	Inhibit AGEs formation	Inhibit AGEs formation	Diabetic Retinopathy	Deng et al. (2021)
Nembutal	*In vivo*	SD rats (300–340 g)	Puerarin 40 mg/kg for 72 h (i.p., n = 5)	Anti-inflammatory	Inhibit ICAM-1 expression	Diabetic Retinopathy	Li, (2007)
Diabetes induced by STZ	*In vivo*	Male Wistar rats (240–260 g)	Puerarin 2, 5 mg/kg for 12 weeks (i.g., n = 20)	Anti-inflammatory	IGF-1 and TNF-α expressions	Diabetic Retinopathy	Yang et al. (2016b)
Diabetes induced by STZ	*In vivo*	Male mice (200–220 g)	Puerarin 50, 100 mg/kg/d for 8 weeks (n = 10)	Anti-apoptosis	Increase Bcl-2 expression (Bcl-2)	Diabetic Cardiomyopathy	Ling et al. (2011)
Diabetes induced by STZ	*In vivo*	Male SD rats (180–240 g)	Puerarin 100 mg/kg/d for 8 weeks (i.p., n = 11)	Inhibit AGEs formation	Inhibit AGEs formation	Diabetic Cardiomyopathy	Ye et al. (2013)
Diabetes induced by STZ	*In vivo*	Male SD rats (200–300 g)	Puerarin 40, 80, 160 mg/kg/d for 12 weeks (i.g., n = 10)	Inhibit RAS System	Inhibit RAS System (Ang-II)	Diabetic Cardiomyopathy	Gao et al. (2017)
IHG	*In vitro*	SCs from the sciatic nerves of neonatal SD rats	Puerarin 10, 25, 50 lmol/l for 48 h	Anti-apoptosis	Inhibit ROS production	Diabetic Peripheral Neuropathy	Chen et al. (2013)
Diabetes induced by STZ	*In vivo*	Male SD rats (6 weeks, 180–220 g)	Puerarin 80, 160, 120 mg/kg for 12 weeks (i.p.)	Anti-inflammatory	MAPK pathway (NO)	Diabetic Peripheral Neuropathy	Zhao et al. (2008)
Diabetes induced by STZ/CCI	*In vivo*	Male SD rats (220–250 g)/CCI	Puerarin 4, 20, 100 nM for 7 days	Anti-inflammatory	NF-κB pathway (NF-kB)	Diabetic Peripheral Neuropathy	Xue et al. (2017)
Diabetes induced by STZ	*In vivo*	SD rats (12 weeks, 107.2–131.88 g)	Puerarin 10, 20, 40 mg/kg for 20 weeks (i.g., n = 20)	Anti-inflammatory	PI3K/Akt pathway	Cognitive Disorders	Chen et al. (2010b)
Diabetes induced by STZ	*In vivo*	Male Wistar rats (210–230 g)	Puerarin 100mg/kg/d for 7 days (p.o., n = 20)	Anti-oxidative stress; Anti-inflammatory	NF-κB pathway (NF-kB)	Cognitive Disorders	Liu J et al. (2014)
Diabetes induced by STZ	*In vivo*	Female C57 mice (3 months, 18–22 g)	Puerarin 25, 50, 100 mg/kg/d for 4 weeks (icv., i.g.)	Anti-oxidative stress	Anti-oxidative stress response	Cognitive Disorders	Hao et al. (2019)

### Diabetic Nephropathy

Some studies discovered that reduction of NAD + profile was induced by hyperglycemia in the podocytes. Following the condition, the expression levels of SIRT1 and peroxisome proliferator-activated receptorγcoactivator-1α (PGC-1α) were decreased, and mitochondrial damage occurred (Xu et al., 2016). *Puerarin* reduced oxidative stress by activating SIRT1. SIRT1 serves as a regulator of Foxo1, *Puerarin* activated downstream pathway of Foxo1 through activating SIRT1, accordingly stimulated the synthesis of PGC-1α, subsequently elevated the anti-oxidant target gene MnSOD and catalase (CAT) by SIRT1/Foxo1 pathway, and driven the anti-oxidant effect, may lead to a reduction of ROS, meanwhile, down-regulation of IL-6, TNF-α in the kidney (Xu et al., 2016). On the other hand, *Puerarin* regulated NADPH oxidase 4 (NOX4) expression through the SIRT1-NF-κB pathway in podocytes. *Puerarin* decreased nuclear factor kappa-light-chain-enhancer of activated β-cells (NF-κB) activation through activating SIRT1, subsequently restrained release to proinflammatory cytokines, and decreased NOX4 expression, the primary enzyme contributing to the increased oxidative stress in podocytes among the different NADPH oxidase (NOX) isoforms, thus reduced oxidative stress (Xu et al., 2016; Li et al., 2017). It also acted directly on nuclear factor-kBp65 (NF-kBp65) and reduced tumor necrosis factor-α (TNF-α) expression by down-regulating NF-kBp65 expression in kidney tissue (Cui and Wang, 2010). Alternatively, it acted directly on intercellular cell adhesion molecule-1 (ICAM-1) and TNF-α and inhibited expressions of ICAM-1 and TNF-α, inhibited non-enzymatic glycosylation of proteins, and relieved oxidative stress inflammatory reaction damage (Pan et al., 2015).

On the other hand, transforming growth factor-β1 (TGF-β1) is one of the probiotic growth factors. A high blood glucose level can induce broad expression of the TGF-β1 gene and protein in the kidney (She et al., 2014). Excessive activation of the TGF-β1/Smad2 signal pathway results in the kidney’s extracellular matrix (ECM) accumulation. Therefore *Puerarin* exerted its anti-diabetic effect by inhibiting the TGF-β1/Smad2 signaling pathway and reducing the accumulation of extracellular matrix in the kidney (She et al., 2014). In addition, *Puerarin* could restrain the protein expressions of TGF-β1 and TGF-β1 receptors (TGF-β-RI) in the kidney tissue of KKAy mice via reducing the expression of α-smooth muscle actin (α-SMA) (Yi et al., 2013). It also inhibited the TLR4/MyD88/NF-kBp65 pathway by up-regulating miRNA-140-5p, thus reducing expression levels of TNF-α, interleukin-1β (IL-1β), interleukin-6 (IL-6), interferon-γ (INF-γ), and TGF-β1 in renal tissues of diabetic rats (Xu X et al., 2020). These changes may inhibit and reverse the epithelial-mesenchymal transition process, thus delaying the occurrence, preventing diabetes-induced renal damage and the development of DN.

Moreover, *Puerarin* can promote autophagy mechanisms through the following two signaling pathways. *Puerarin* modulated ERS/autophagy crosstalk by regulating activated extracellular signal-regulated kinase (*p*-ERK), persistent ERS activated the *p*-ERK-eukaryotic translation initiation factor 2α (eIF2α) signaling pathway, activating transcription factor 4 (ATF4) is then up-regulated in response to eIF2α phosphorylation. CHOP and Beclin-1 were activated through regulating of the PERK/eIF2α/ATF4 signaling pathway, up-regulated the levels of autophagy markers Beclin-1, microtubule-associated protein light chain 3 II (LC3II), and autophagy-related 5 homolog (Atg5), and down-regulated the level of p62, thus resulted in the autophagy response (Xu Y et al., 2020). It also protected against podocyte injury by restoring the autophagic activity via the AMPK/SIRT1 pathway. *In vitro* and *in vivo* experiments demonstrate, *Puerarin* stimulated SIRT1 expression in podocytes to deacetylate liver kinase B1 (LKB1) and then phosphorylated AMPK-mTOR pathway to induce autophagy (Li et al., 2020).

Advanced glycation end products (AGEs) and RAGE play an essential role in developing diabetic nephropathy. *Puerarin* could protect the renal tissue from the impairment of hyperglycemia and AGE by decreasing AGEs contents and inhibiting the expression of RAGE mRNA in the kidney, which may due to decrease blood glucose directly, or reduce AGEs formation by inhibiting oxidative stress, aldose reductase activity, and, so on (Shen et al., 2009). Furthermore, *Puerarin* also attenuates eNOS expression in glomerular endothelial cells (Zhang et al., 2015).

Together, *Puerarin* can achieve inhibitory non-enzymatic glycosylation of the protein, alleviate oxidative stress or inflammatory response damage, and promote autophagy response through the above pathways, thus protecting diabetes-induced kidney damage and delaying the occurrence and development of DN.

### Diabetic Macroangiopathy

Hyperglycemia could cause a non-classic inflammation response in the vascular endothelium and contributes to the inflammation response through the aggregation of intracellular ROS (Lian et al., 2019). So there is a correlation between DM and the occurrence of oxidative stress products and inflammatory factors in a high-glucose environment. *Puerarin* inhibits the occurrence of oxidative stress and inflammatory response mainly through the following signaling pathways.

ROS in vascular the primary source is the NADPH oxidase (NOX family). ROS is a critical upstream activator of the NF-kB pathway (Li et al., 2016), which consequently increases the expressions of lectin-like oxidized low-density lipoprotein receptor-1 (LOX-1), ICAM-1, and E-selectin, and Nlrp3 inflammasome activation (Li et al., 2016). High mobility group box 1 (HMGB1) release is a downstream product of Nlrp3 inflammasome activation (Lian et al., 2019). Thus, NF-kB, the critical transcription factor in regulating molecular adhesion expression, plays an essential role in DM. *Puerarin* inhibited NADPH oxidase 2 (NOX2) and NOX4 expressions in vascular smooth muscle cells (VSMCs) through regulating NF-kBp65 to suppress oxidative stress and expressions of cell adhesion molecules (Li et al., 2016). It also attenuated IKKb phosphorylation and effectively blocked NF-kB activation by inhibiting NF-kBp65 phosphorylation and decreased TNF-α and IL-6 production by inhibiting IKKb/NF-kB activation (Huang et al., 2012). In addition, this is a new protection mechanism of *Puerarin* that inhibited NF-kB activation by suppressing oxidative stress, subsequently inhibited Nlrp3 inflammasome activation, attenuated TXNIP-NLRP3 binding, decreased subsequent Caspase-1 activation, and decreased the release of HMGB1 (Lian et al., 2019).

Moreover, *Puerarin* also stimulated endothelial nitric oxide synthase (eNOS) phosphorylation and nitric oxide (NO) production via activation of an estrogen receptor-mediated PI3K/Akt and CaMKII/AMPK dependent pathway. It attenuated phosphorylation of IRS-1 at S307 and effectively ameliorated the tyrosine phosphorylation of IRS-1, which activated PI3K, subsequently activated PI3K phosphorylates and activated downstream target Akt which directly phosphorylated eNOS at Ser1177, leading to increased production of NO(Hwang et al., 2011). (Figure 3)

*In vitro*, *Puerarin* disrupted the phosphorylation and membrane translocation of PKCβ2 as well as Rac1, p47phox, and p67phox subunits and NADPH oxidase activation in VSMCs may exert inhibitory effects on high-glucose-induced VSMC proliferation via interfering with PKCβ2/Rac1-dependent ROS pathways (Zhu et al., 2010).

In summary, *Puerarin* inhibited inflammatory response and oxidative stress via inhibiting NF-kB activation, and it inhibited oxidative stress via ameliorating PI3K/Akt, CaMKII/AMPK, and PKCβ2/Rac1 pathway. Accordingly, *Puerarin* ameliorated IR-associated endothelial dysfunction.

### Diabetic Retinopathy

DR is an alteration of diabetic microangiopathy in the specific environment of the fundus. Long-term chronic hyperglycemia causes oxidative stress, inflammatory response, and non-enzymatic glycosylation of proteins, which promotes apoptosis and accelerates the onset of DR. *Puerarin* protects retinal function through the following pathways.

In response to high glucose toxicity, the activity of ROS is increased, causing phosphorylation of retinal capillary endothelial cells in JAK2/STAT3, thereby increasing the expression of vascular endothelial growth factor (VEGF). Thus, the mechanism of the *Puerarin* effect is hypothesized to be due to inhibition of the phosphorylation of JAK2/STAT3, thereby reducing the expression of VEGF and the inflammation of the retina, and that results in preventing the occurrence of DR (Cai et al., 2017). Furthermore, *Puerarin* decreased the expression levels of retinal vascular endothelial growth factor (VEGF) and IL-1β through activating nuclear factor-E2 related factor2 (Nrf2)/HO-1 signaling pathway (Zhang and Li, 2019) and inhibiting the Nrf2/ERK signaling pathway (Zhang and Wang, 2019), thus, reduced the inflammatory response and inhibiting oxidative stress of retinal tissue.

The Fas/FasL system is considered the primary signal transduction pathway to mediate apoptosis, and it may affect and strengthen the apoptosis process mediated by ONOO- (Hao et al., 2010; Hao et al., 2012). Thus, *Puerarin* could protect retinal pigment epithelial cells (RPE) of diabetic rats through inhibiting inducible nitric oxide synthase (iNOS) up-regulation and ONOO-generation mediated through Fas/FasL signal pathway (Hao et al., 2010; Hao et al., 2012).

The prolonged hyperglycemic state can induce the production of AGEs. *Puerarin* inhibited the productions of AGEs-modified proteins and their accumulation in the retina by inhibiting the non-enzymatic glycosylation of proteins to improve DR. Components of flavonoid structures can capture dicarbonyl compounds (key intermediates in AGEs formation) and form adducts, thus exerting an inhibitory effect on AGEs formation, and it is speculated that the mechanism of action of total flavonoid extracts of *Pueraria* to inhibit AGEs levels may be related to this (Liu et al., 2018; Deng et al., 2021).

In addition, *Puerarin* raised the expression of insulin-like growth factor-1 (IGF-1) and decreased the expressions of TNF-α, ICAM-1, IL-1β, and IL-6 in the retina to protect the function of the retina (Li, 2007; Yang et al., 2016b; Deng et al., 2021).

In conclusion, *Puerarin* could protect RPE cells and the function of the retina by reducing the inflammatory response, inhibiting oxidative stress and apoptosis of retinal tissue, inhibiting nonenzymatic glycosylation reactions of the proteins.

### Diabetic Cardiomyopathy

Chronic hyperglycemic environments promote oxidative stress and the release of inflammatory factors through processes such as glycosylation, thus damaging the myocardium. *Puerarin* may reduce myocardial damage through the following pathways.

On the one hand, *Puerarin* increased Bcl-2 expression in the myocardium, afterward suppressed permeabilization of the mitochondrial inner membrane to Cyt-C, subsequently reduced the release of Cyt-C, inhibited the formation of apoptotic bodies, subsequently regulated activation of the Caspase-3, are suggested to be the mechanisms responsible for *puerarin’s* anti-apoptotic effect against diabetic cardiomyopathy in STZ induced SD rats (Ling et al., 2011). (Figure 2)

Moreover, it also reduced RAGE expression at the mRNA level by reducing blood sugar, subsequently reduced AGE-RAGE binding, reduced the myocardial oxidative stress and the inflammatory response. Thus injury was relieved by AGEs in diabetic rats (Ye et al., 2013).

For another, AT1R mediates almost all cardiovascular effects of angiotensin-II (Ang-II). *Puerarin* decreased myocardial AT1R protein expression and reduced cardiac Ang-II levels in diabetic rats. On the one hand, it inhibited renin-angiotensin system (RAS) activation and suppressed cardiomyocyte hypertrophy and fibrosis, thus improving cardiac systolic and diastolic functions. On the one hand, it inhibited TNF-α and IL-1β expressions, suppressed Caspase-9 activation, inhibited inflammatory factor release and cardiomyocyte apoptosis, thereby protecting the myocardium. In addition, *Puerarin* could improve IR by decreasing Ang-II levels (Zhao et al., 2008; Chen et al., 2013; Gao et al., 2017).

### Diabetic Peripheral Neuropathy

DPN is one of the most prevalent and debilitating microvascular complications of diabetes, affecting at least 50% of people with diabetes (Xue et al., 2017). Schwann cells (SCs) are the most critical myelinating cells of the peripheral nervous system. Hyperglycemia-induced abnormalities of SCs could cause a cause of demyelination of nerve fibers, reduction of regeneration capability in peripheral nerves, and axonal atrophy, resulting in the development of DPN(Xue et al., 2017). Therefore, to SCs, *Puerarin* significantly inhibited glucose fluctuation-stimulated ROS production and mitochondrial depolarization in SCs, subsequently suppressed mitochondrial dysfunction, down-regulated the expression of proapoptotic factors (e.g., Bax), and up-regulated the expression of anti-apoptotic factors (e.g., Bcl-2), which subsequently suppressed Caspase-3 activation and PARP cleavage in SCs, thus inhibited SCs apoptosis (Xue et al., 2017). In addition, *Puerarin* activated T-type Ca2+ channel and mitogen-activated protein kinases (MAPK) signaling by up-regulating NO level, which up-regulated the expression of the calcitonin gene-related peptide (CGRP) gene to enhance the antagonistic effect of CGRP on endothelin-1 (ET-1), improved endure blood supply (Chen et al., 2010b). The anti-inflammation effect of *Puerarin* might be related to the suppression of spinal NF-kB activation or cytokines upregulation (Liu M et al., 2014).

Moreover, *Puerarin* significantly inhibited the proinflammatory response and oxidative stress in the cerebral cortex and hippocampus by activating PI3K/Akt signaling pathway (Hao et al., 2019) and inhibiting NF-kB activation (Liu et al., 2016), which determines its cognitive protection in diabetes (Yang et al., 2015).

## Research Conclusion

Diabetes is a non-communicable metabolic disease characterized by chronic hyperglycemia (Meng et al., 2017; Niu et al., 2017). Severe or persistent hyperglycemia-induced a series of diabetic complications cannot be ameliorated by antidiabetic agents, let alone their adverse side effects, including hypoglycemia, gastrointestinal reactions, liver damage, and lactic acidosis. All these raise concerns over their safety and efficacy in diabetic patients (Srivali et al., 2015). Currently, more proposals of applications of alternative treatment for diabetes and its complications to counteract these side effects have been put forward, of which RP, the most frequently and long-term used TCM, has become a hotspot. The pharmacological properties of Puerarin, a major active component of RP, have been recently uncovered (Cao et al., 2006). *Puerarin* has been shown to exert antidiabetic effects of reducing blood glucose and improving diabetes complications in patients (Wong et al., 2011). It has been proven to promote β-cell neogenesis and inhibit apoptosis, enhance the insulin receptor signaling, boost glucose transport and uptake, and suppress hepatic gluconeogenesis through multiple approaches, including activation of GLP-1R and PI3K/Akt signalings and inhibition of ROS production and Caspase/AIF apoptotic pathway in the pancreas, enhancement of GLUT4 delivery and PPAR receptor expression alongside increased fatty acid oxidation in skeletal muscle and adipose tissue, and PI3K/Akt activation in the liver. Thus, insulin secretion is restored to improve IR to lower blood glucose. As for diabetic complications, *Puerarin* has been proven to significantly delay their occurrence and progression via eliminating excessive nonenzymatic glycosylation, oxidative stress, and inflammatory response and suppressing apoptosis caused by chronic hyperglycemia.

However, limitations on current diabetic research are apparent, despite significant findings of molecular mechanisms for *Puerarin’s* antidiabetic effects. Most clinical studies in this field merely show the low-to-moderate level of evidence, and large-sample randomized controlled studies are urgently needed to offer convincing conclusions. Besides, the effective dose and safety of *Puerarin* in each type of diabetic complication have not been determined, which calls for more *in-vivo* and *in-vitro* experiments and validation by clinical studies or large-sample cohort studies. Moreover, current studies only ascertain the limited efficacy of *Puerarin* in the treatment of diabetic patients with complications. Overall, *Puerarin* is a promising new treatment for diabetes and its complications. Further studies into this topic are warranted.

## Glossary

1-A: 1-Adrenaline
α1-AR: α1-Adrenergic Receptors
α-SMA: Alpha-Smooth Muscle Actin
α1A-AR: α1A-Adrenergic Receptors
β-EP: β-Endorphin
Bax: Bcl-2-Associated X
Bcl-2: β-cell lymphoma-2
ACC: Acetyl Carboxylase
AGEs: Advanced Glycation End Products
Akt/PKB: Protein Kinase B
Akt1: Protein Kinase Bα2
AMPK: AMP-Activated Protein Kinase
Ang-Ⅱ: Angiotensin II
ATF4: Activating Transcription Factor 4
Atg5: Autophagy-Related 5 Homolog
CAT: Catalase
CD36/FAT: Fatty Acid Translocase
CGRP: Calcitonin Gene-Related Peptide
CPT-1b: Carnitine Palmitoyltransferase-1b
Cyt-C: Cytochrome C
DM: Diabetes Mellitus: Diabetic Macroangiopathy
DPN: Diabetic Peripheral Neuropathy
DN: Diabetic Nephropathy
DM: Diabetes Mellitus: Diabetic Macroangiopathy
DR: Diabetic Retinopathy
DC: Diabetic Cardiomyopathy
eIF2α: Eukaryotic Translation Initiation Factor 2α
ECM: Extracellular Matrix
eNOS: Endothelial Nitric Oxide Synthase
ET-1: Endothelin-1
FBG: Fasting Blood Glucose
Foxo1: Forkhead Box Transcription Factor O1
GLP-1R: Glucagon-like Peptide-1 Receptor
GLUT4: Glucose Transporter 4
GSK-3β: Glycogen Synthase Kinase-3β
G6pase: Glucose-6-Phosphatase
HbA1c: Glycated Hemoglobin
HMGB1: High Mobility Group Box 1
ICAM-1: Intercellular Cell Adhesion Molecule-1
IDF: International Diabetes Federation
IGF-1: Insulin-like Growth Factor-1
IL-6: Interleukin- 6
IL-1β: Interleukin-1β
INF-γ: Interferon-γ
IMCLs: Intramyocellular Lipids
iNOS: Inducible Nitric Oxide Synthase
InsR: Insulin Receptor
IRS-1: Insulin Receptor Substrate-1
IR: Insulin Resistance
LC3II: Microtubule-Associated Protein Light Chain 3 II
LKB1: Liver Kinase B1
LOX-1: Lectin-like Oxidized Low-Density Lipoprotein Receptor-1
MAPK: Mitogen-Activated Protein Kinases
MDA: Malondialdehyde
NF-kBp65: Nuclear Factor-kBp65
NF-κB: Nuclear Factor Kappa-Light-Chain-Enhancer of Activated β-Cells
NO: Nitric Oxide
NOX: NADPH Oxidase
NOX4: NADPH Oxidase 4
NOX2: NADPH Oxidase 2
Nrf2: Nuclear Factor-E2 Related Factor2
PARP: Poly ADP-Ribose polymerase
PDX-1: Pancreatic Duodenum Homeobox-1
PDE3: Phosphodiesterase 3
PEPCK: Phosphoenolpyruvate Carboxykinase
*p*-ERK: Activated Extracellular Signal-Regulated Kinase
PGC-1α: Peroxisome Proliferator Activated Receptorγcoactivator-1α
PI3K: Phosphatidylinositol 3 Kinase
PLC-PKC: Phospholipase C-Protein kinase C
PPARα: Peroxisome Proliferators-activated Receptors α
PPARγ: Peroxisome Proliferators-activated Receptors γ
PKB: Protein Kinase B
PTP-1B: Protein Tyrosine Phosphatase-1B
RAS: Renin-Angiotensin System
ROS: Reactive Oxygen Species
RP: Radix Puerariae
RXR: Retinoic Acid Receptor
RPE: Retinal Pigment Epithelial Cells
SCs: Schwann Cells
SIRT1: Sirtuins1
SIRT3: Sirtuins 3
SOD: Superoxide Dismutase
SOD2: Superoxide Dismutase 2
TCM: Traditional Chinese Medicine
TGF-β1: Transforming Growth Factor-β1
TGF-β-RI: Transforming Growth Factor β I Receptors
TNF-α: Tumor Necrosis Factor-α
TXNIP: Thioredoxin-Interacting Protein
UCP2: Uncoupling Protein 2
VSMCs: Vascular Smooth Muscle cells
VEGF: Vascular Endothelial Growth Factor
